# Review of Court Cases on AdvanceCare Planning: Identifying Iimplementation Issues and Challenges

**DOI:** 10.1093/geroni/igaf122.808

**Published:** 2025-12-31

**Authors:** Megumi Inoue, Meng-Hao Li, Yifan Lou, Nina Buckley, Lana El-Khatib, Isabelle Davis

**Affiliations:** George Mason University, Fairfax, Virginia, United States; George Mason University, Fairfax, Virginia, United States; Virginia Commonwealth University, Richmond, Virginia, United States; George Mason University, Fairfax, Virginia, United States; George Mason University, Fairfax, Virginia, United States; George Mason University, Fairfax, Virginia, United States

## Abstract

**Introduction:**

Advance directives (ADs) are a critical component of advance care planning (ACP), formally documenting healthcare preferences to ensure an individual’s end-of-life care wishes are respected and followed. However, if ADs are incomplete or improperly completed, legal disputes may arise, potentially leading to unsatisfactory or unforeseen outcomes. This study analyzed court records related to ACP to identify issues in its implementation.

**Design:**

Data for this study were extracted from the Caselaw Access Project (CAP) database, the largest publicly available dataset of legal documents. A total of 291 court reports published between 1980 and 2019 in the U.S. that contained the keywords “advance directive” or “advance care planning” were extracted for analysis. A thematic analysis was conducted to identify the key issues that led to court cases.

**Results:**

After removing unrelated cases, such as those concerning asset wills, 79 cases were included in the analysis. The major issues identified were the conflicts among stakeholders over appropriate treatments compounded by the absence of ADs or related ACP measures (n = 53), physician noncompliance with existing ADs (n = 14), and challenges to the legitimacy of ADs (n = 12).

**Conclusion:**

The findings highlight the importance of having an AD in place as a legal document to safeguard individual wishes and prevent family and/or family-physician conflicts. Our study also found that even with an AD, goal-concordant care isn’t guaranteed, leading to legal cases. This emphasizes that discussions with family and healthcare providers, ensuring awareness and understanding of ACP, are as important as, if not more than, the AD itself.

